# Technology-Enhanced Health Promotion for College Students: A Seed Development Project

**DOI:** 10.3390/nursrep11010014

**Published:** 2021-03-01

**Authors:** Carol A. Fackler, Nancy Baugh, Ann A. Lovegren, Carol Nemeroff, Janet Whatley Blum

**Affiliations:** 1 School of Nursing, University of Southern Maine, Portland, ME 04104, USA; 2 School of Nursing and Population Health, University of New England, Portland, ME 04103, USA; nbaugh@une.edu; 3 CVS MinuteClinic, South Portland, ME 04106, USA; ann.lovegren@minuteclinic.com; 4 Renaissance College, University of New Brunswick, Fredericton, NB E3B 1M7, Canada; carol.nemeroff@unb.ca; 5 School of Health Sciences, Merrimack College, North Andover, MA 01845, USA; blumj@merrimack.edu

**Keywords:** health promotion, college student health promotion, technology-based health promotion, obesity prevention, healthy lifestyle, college transition

## Abstract

Obesity is an issue for young adults in the U.S. This population is particularly vulnerable to weight gain as they move from adolescence to young adulthood, especially as they transition from high school to college. Adopting a health promotion approach, a university-based cluster of researchers, community advocates, and a technology partner embarked on a two-year seed development project that focused on development, implementation, and evaluation of a web-based healthy lifestyle intervention for college students. Using a mixed-method design, two convenience samples of residential university students were recruited to participate in a 4-week intervention called Eat, Move, Live, in which they interacted with a newly-created comprehensive website about management of a healthy lifestyle. Participants’ post-intervention readiness for change increased by 15% (eating and life balance behaviors) to 23% (moving behaviors). Participants reported increased awareness of eating behaviors, and feelings of engagement in tracking their fruit and vegetable consumption. Findings suggest that technology may be utilized to enhance the effectiveness and efficiency of achieving students’ individual goals related to healthy living. These preliminary findings have implications for increasing the development and implementation of technological approaches to health promotion for young adult students.

## 1. Introduction

Creative approaches to health promotion can be supported in a number of ways. At one university in Maine, monies from the Maine Economic Improvement Fund (MEIF) were used to support a multidisciplinary group of faculty, community partners, and students to achieve outcomes related to the use of information technology to promote a healthy lifestyle in college students. A “research cluster” formed for this project combined the expertise of academics and practitioners in nursing; exercise, health, and sport science; social and behavioral science; nutrition; and population health. A technology industry partner from the metropolitan community in which the university resides provided guidance in the development of the project and technical consultation to university students, who developed the IT prototype used as the intervention website. 

The Maine Economic Improvement Fund (MEIF) was established by the Maine Legislature in 1997 to advance research and product innovation in the University of Maine system and to respond to targeted community needs. Requirements by the MEIF for information technology initiatives stipulate that there must be involvement by a multidisciplinary team that includes community partners and a local member of the technology industry. This project’s multidisciplinary research cluster was interested in developing a web-based lifestyle management intervention for college students. The cluster included university faculty from Nursing; Exercise, Health and Sports Science; and Social and Behavioral Sciences as well as an advanced practice nurse in College Health Services. Community participation included representatives from the local chapter of the American Heart Association—an independent nutrition consultant—and a population health specialist from the state’s Center for Disease Control. The technology industry partner was a local innovative web design company; this partner’s role was to advise the project in web design. As the project began to unfold, significant contributions to the design of the two pilot studies’ intervention were made by university students working with the research faculty.

Adopting a population health approach, the research cluster envisioned that healthy lifestyle management in college students could positively influence outcomes of heart disease and type 2 diabetes [1]. Obesity, recognized by the U.S. Centers for Disease Control (CDC) as a risk factor for morbidity from heart disease and diabetes [2], was the focus of the intervention in the studies. Defining the success of this project was based on the evaluation of four outcomes: (1) physical and psychological outcomes of the intervention; (2) evaluation of the IT prototype; (3) student engagement; and (4) ongoing evaluation of cluster member collaboration during the planning, implementation, and evaluation of the project.

Chronic diseases such as heart disease, diabetes, cancer, and kidney disease, continue to be the leading cause of morbidity and mortality in the U.S. [1]. The cost to care for chronic illness accounts for 90% of the country’s health care expenditures [1]. Even more alarming is the increased incidence of chronic disease occurring in adolescents and young adults worldwide. Diseases associated with the obesity epidemic, including hypertension, type 2 diabetes, metabolic syndrome, sleep apnea, kidney disease, and fatty liver disease are becoming leading causes of morbidity in this population [3,4,5,6,7]. In adolescents, the prevalence of chronic diseases such as diabetes and hypertension has been reported to be higher than in other age groups. In a representative sample of the U.S. population, researchers reported a prevalence of diabetes in adolescents of 0.72%, while the prevalence was only 0.13% in the 6–11 year old age group in this study. Differences in prevalence for hypertension was 0.46% in the adolescent group, and 0.04% in the 6–11 year old age group [8]. 

Addressing obesity early in life may prevent the development of other chronic illnesses, and thereby decrease the morbidity and mortality associated with longstanding chronic disease. The CDC reports that the current prevalence of obesity among younger adults, age 20–39, is 40% [9]. In 2012, the American College Health Association reported 34.3% of American college students as being overweight or obese [10]. Current research continues to demonstrate that students transitioning from high school to college are particularly vulnerable to weight gain during their college years [11,12,13]. There is growing evidence that chronic diseases such as type 2 diabetes, hypertension, and hyperlipidemia are commonly seen in the college age population and can be directly linked to weight gain [14,15].

The college years provide a unique opportunity to promote and shape healthy behaviors; however, few studies have examined how to effectively engage college students in achieving and maintaining a healthy lifestyle and there is little application of evidence-based practice on campuses nationwide. College students commonly use the Internet for seeking health information and increasingly use smart phones to access health information and interventions [16,17,18]. Interventions that are technology based are associated with greater acceptance among this population when compared to more traditional intervention methods (e.g., counseling) [19]. Web-based programs for college students have successfully impacted smoking cessation, excessive alcohol intake, and marijuana use [20,21,22,23,24,25]. And the use of varied internet-based technologies in health-related interventions with this population is proving to be effective, including Facebook [26] and text messaging [27,28]. However, there is little published evidence on the use of internet-based interventions in the areas of weight loss, obesity prevention, or healthy lifestyle management for college students. The purpose of the pilot studies within this two-year seed development project was to develop, implement, analyze, refine, and retest a web-based comprehensive lifestyle management intervention for college students targeted toward obesity management and prevention, with a primary assessment focus on behaviors related to eating. In addition, the research cluster identified five outcomes to address and assess in the seed development project: 

### 1.1. Outcome 1

Student research participants are able to use the technology by which the lifestyle management intervention is delivered. 

### 1.2. Outcome 2

Student research participants understand and are motivated by the information provided through the technology-driven lifestyle management intervention. 

### 1.3. Outcome 3

Student research participants engage in the interactive aspects of the technology delivering the lifestyle management intervention. 

### 1.4. Outcome 4

Some student research participants move from one stage of readiness for change [29] to another at the end of the comprehensive intervention. 

### 1.5. Outcome 5

The Research Cluster collaborates in meaningful ways toward achieving the successful completion of each phase of the two-year seed development project. 

## 2. Materials and Methods

Using a mixed-method design, two convenience samples of residential university students were recruited to participate in two separate 4-week interventions. Twenty-two (22) participants were enrolled in the first pilot study; also, twenty (20) participants were enrolled in the second pilot study. Student participants interacted with a newly-created comprehensive website containing information and activities related to management of a healthy lifestyle spanning three broad areas: eating, moving (physical activity), and life balance (including stress management), branded as ‘Eat, move, live!’. The intervention took place during 2013–2015. Recruitment events included frozen yogurt socials in student residence halls, as well as tabling in both student residence halls and the campus dining center. For those students expressing interest, follow-up phone calls by members of the research team determined eligibility by assessing potential participants’ readiness for change [29] based on four questions determining readiness in each of the areas of eating, moving, and life balance. Those students who indicated in any of the three areas that they were in a ‘preparation’ or ‘action’ stage of readiness were considered eligible to participate in the study. Those who indicated that they were in a ‘precontemplation’ or ‘maintenance’ stage of readiness in all three areas were deemed not eligible to participate. 

For each pilot study, the intervention consisted of providing access to a website designed to promote a healthy lifestyle. The website for the first pilot study included basic information on healthy eating, physical exercise, and stress reduction. The website for the second pilot study was more sophisticated in design and content, additionally including information on effective goal setting, and videos and links to evidence-based recommendations for healthy living. After setting personal goals for a healthy lifestyle, and then accessing the website to review information on eating, moving, and life balance, study participants were asked to track their fruit and vegetable consumption weekly, and to interact with team members and fellow participants via social media (Facebook, Twitter) as part of the intervention. University Institutional Review Board approval was obtained for each of the two pilot studies. Informed consent was obtained from all subjects involved in the studies. For each study, an orientation session included an informed consent procedure, followed by an introduction to the website, explanation of self-tracking eating behaviors, and encouragement to use social media during the 4-week intervention period. Quantitative data collected at the beginning and end of each pilot intervention included participants’ readiness for change (Outcome 4), self-reported height and weight, and food consumption frequency for fruits and vegetables (Outcomes 2, 4). During the intervention, participants were asked to complete and return a survey at the end of each of the four weeks about their level of participation in the intervention (Outcomes 1, 2, 3). Questions assessed the frequency of accessing the website, what information was read, how much self-tracking of fruit and vegetables was done, and whether or not social media was used. At the end of each pilot study, qualitative data were collected to explore student participants’ perceptions regarding use and comprehension of the web-based intervention, as well as their level of engagement and ease of interaction with the intervention website (Outcomes 1, 2, 3). The qualitative data was obtained through individual participants’ written narrative summaries in response to specific questions, as well as focus group conversations.

## 3. Results

Post-intervention narrative data were obtained from 12 out of 22 students in the first study, and 13 out of 20 students in the second study. Those students willing to disclose why they ceased participation cited time constraints related to their academic work as the reason for attrition. Focus group participation was limited, with less than half of each pilot study’s participants participating in a post-intervention focus group. However, feedback from the focus group conversations was consistent with the findings of the written narrative summaries.

Across all participants in both pilot studies, there was no change in self-reported weight. While there was no difference in readiness for change from pre- to post-intervention in the first pilot study, differences were noted in the second study. Under the “Eating” portion of the intervention, 3 (23.1%) participants increased their readiness level; under “Moving”, 3 (23.1%) participants increased their readiness level; under “Life Balance”, 2 (15.4%) participants increased their readiness level. Given the small amount of data available related to food frequency, reliable statistical analyses could not be completed; however, the impact of the tracking process is discussed below.

Participants completed anonymous written summaries of their experience with the web-based intervention in answer to questions about ease of use, usefulness, knowledge gained, motivational content, and making the website more user friendly. The majority of the 12 respondents (11 out of 12) in the first pilot study found the website easy to use. Those who found it useful stated the following reasons for this view: there was information about how many servings to eat; it reminded them to keep track [of what they ate] which influenced their behavior; it was good content for someone who does not already know a lot about this topic. 

Ten of the 12 participants in the second pilot study identified components of the intervention they could learn from. Learning was described in terms of general skills such as setting goals and self-tracking. Others noted learning to be the result of helpful information that was available. Eleven of the 12 participants identified components of the intervention that were engaging and maintained their interest, including information provided on the website; communication aspects such as self-tracking, Facebook posts, and email reminders; and the stylistic aspects of the website itself. 

In describing the knowledge gained from participating in the intervention, the majority of those responding in the first pilot study noted that the process of self-tracking their eating behaviors increased awareness about their own behavior, and the effectiveness of keeping track of that behavior. In addition, the content of the information on the website provided participants with an awareness of ideal behavior for comparison. Participants also noted that the information gained from the website, and their tracking of their eating behaviors are what they found engaging and motivating.

Across both pilot studies, there were suggestions for improving the project’s web-based intervention. Some of these suggestions were incorporated in the website revision conducted between the first and second pilot studies; others were not within the technological capabilities of the project’s resources during the seed project period. Suggestions made included: making the tracking feature a mobile phone application; creating the ability for users to add goals to the tracking feature; creating the ability for users to track progress/change over time; creating the ability for users to compare their tracking results to those of others, perhaps even in a competitive way; increased opportunities for interactivity with the website features, such as the ability to input goals and track progress directly; the ability to receive instant feedback; and the ability to receive individualized feedback and get redirected to relevant information and resources.

Specific suggestions regarding content that would be useful to add to the web-based intervention included information about: portion sizes; recipe links; allergies; and how to read labels. A few participants noted that specific tools would be helpful, including a calorie counter or a nutritional calculator. 

The first pilot study focus group conversation revealed that participating students were satisfied with information on the website, and with the available self-tracking feature of the intervention. Suggestions offered to improve the intervention included: creating the ability for participants to set goals and track progress; consideration of an application to use for tracking; and tailoring the content on the website to that which is most helpful to college students who did not have a lot of money to buy food, to buy produce, or to cook on their own. There was a lot of discussion about working with the university cafeteria to promote eating in a healthier way.

In the second pilot study focus groups, participants noted that while they found the information on the website interesting, many of them focused their study of the website on those areas related to the goal they set (related to “Eating” for example), not reviewing other aspects of the website, or spending less time on those aspects (related to “Moving” and “Life Balance”). Participants noted how helpful they found the self-tracking feature of the intervention, with participants in the first focus group noting in particular that, through tracking, they realized what their actual eating habits were. 

Despite the research cluster’s encouragement to use social media during the four-week intervention, participants in the second pilot study’s focus groups revealed they did not use Twitter; and only those who already regularly used Facebook noted that they paid attention to it in this study. Some participants who did not actively participate in using Facebook noted that they nevertheless followed the posts that were put there by members of the research team. Some also stated that they never saw the emails sent out at the beginning of each week of the four-week intervention. When asked if texting would have been helpful for reminders by the research team, the participants in the first focus group did not want to be texted, while those in the second thought texting would be a good way of communicating. In summary, participants were very clear about how their individual preferences influenced their engagement with aspects of the website intervention, as well as the social media support. However, those preferences differed across individuals.

## 4. Discussion

It was clear from the outset that neither pilot study would allow for a large enough sample to make any inferences about physical or psychological outcomes; therefore, most of the data collected was qualitative rather than quantitative. The qualitative data yielded important insights regarding the robustness of the intervention website in the second pilot study, including what was informative and engaging about the intervention, and how the intervention could better use technology in future work. It was clear from the qualitative data that improvements made based on the first pilot study enhanced the intervention website’s effectiveness in the second pilot study.

Participants liked the IT prototype (the website) utilized for the second intervention, suggesting that more interactive components be added, including mobile applications. One of the positive points in the feedback was the helpfulness of embedded links to sources of lifestyle management information (e.g., from the Centers for Disease Control [CDC]), which ‘validated’ for some participants the information on the website. This is consistent with other research findings noting that college students consider health organization and governmental websites as credible sources of information [30,31].

Perhaps the most insightful feedback was the influence of interacting with the website on participants’ engagement, in a variety of ways, with healthy lifestyle management. The influence was multifactorial and included (self-reported): increased awareness of eating behaviors leading to increased attention to food choices; creation of interest in self-initiated searches for more information on healthy living; and the use of tools that the participants found to put into practice what they were learning on the intervention website (e.g., the Fitbit app). Web-based eating advice continues to be reported as effective in research with college students, with a recent study on a brief web-based nutrition intervention for college students demonstrating high engagement, acceptability, and satisfaction by participants [32]. 

Participants who used the self-tracking tool included in the intervention reported being more attentive to their behaviors. This is consistent with literature that discusses the positive influence self-monitoring has on behavior. For example, self-weighing has been reported to improve weight outcomes in those involved in weight loss or weight maintenance interventions [33]; and self-monitoring daily step counts via pedometers or accelerometers has been shown to increase physical activity in patients with cardiovascular disease [34]. 

Though not surprised at the attrition rate in both pilot studies, the research team was disappointed with both that and the feedback from participants that they had engaged with the website early in the four-week second pilot intervention, but then did not interact with the intervention after that. The inability to sustain participation in the intervention is consistent with recent literature on weight loss interventions. In a systematic review of 16 randomized controlled trials with participation by adults in a technology-assisted weight loss intervention, there was 20% or greater attrition rates in a majority (9/16) of the studies reviewed; in three of the studies, the attrition rate was 40% or greater [35].

Ongoing evaluation of the cluster members’ collaboration has yielded a lot of insight into disciplinary approaches to both research questions and research methodology, team dynamics, communication challenges in working as part of a large research team, and team member strengths and weaknesses in conducting a two-year, two-pilot seed development project. Feedback among research team members was honest and constructive, and members aimed for professional growth as an outcome. 

There were a number of limitations to this project. In the first pilot study, one of the major limitations was the inability to engage computer science students in the project who had sufficient knowledge and skills to develop a website that was engaging as well as informative. The team had hoped to involve students as much as possible in creating the intervention, but as a result of this limitation, participants in the first pilot study were offered only very basic information and ways to engage (e.g., through self-tracking that was created in a Word document and sent via email to the research team). During the development of the first pilot study, very few student hours were used to develop the website due to lack of interest on the part of the computer science student community. During the second pilot study, two more advanced undergraduate students were recruited who not only had an interest in this IT-related project, but also the prior knowledge and skills to develop an engaging website and create a plan for social media engagement.

Recruitment was another major limitation of the project, in both studies. Even using student members of the cluster to engage potential participants (being closer in age to those potential participants than the faculty on the research team), it was very difficult to recruit a sizable number of participants for either pilot study. Despite expressed interest by approximately 50 students towards each pilot, time limitations and other factors intervened resulting in only 20–22 students being enrolled in each of the pilot studies. Of those enrolled in each study, many were then lost to attrition. Of those who divulged why they chose to stop participating, students cited time limitation as the major reason; they had just too much work to do as students. It is possible that encouraging students to first interact with the life balance (stress and time management) domain might improve engagement and decrease attrition in future interventions.

A number of recommendations for working with the college student population can be made based on the results of this project, including implications for practice, education, research, and policy. College students should be encouraged and given opportunities to engage in health lifestyle management, using technology when possible. When sharing information, it is important that the information used is identifiable as being evidence-based. Providing information, no matter how it is delivered, through links to credible scientific sources, will help validate the information for students. 

It is important to educate college administrators and staff on the use of technology in engaging college students to have positive experiences when it comes to managing their health. For any activity or event in this area, it is important that college staff understands the value to college students of tracking their progress and receiving real time feedback. Recognizing that college students may perceive barriers to participating in healthy lifestyle management due to the time needed for academic work, every effort should be made to create interventions and health promotion activities that are tailored to individual needs and preferences. It may be useful to focus first on stress- and time-management strategies, to remove barriers to other activities. Technology may be utilized to enhance the effectiveness and efficiency of achieving individual goals related to healthy living.

## 5. Conclusions

College students are an important population to study. It is notable that this population is a difficult one to recruit; however, once involved in a project, some college students will become engaged and motivated to act. College students today like feedback in real time, and they are comfortable with the use of technology; however, one size does not fit all. Different students like different kinds of technology; tailoring interventions to preferred technology will make it more likely that this generation will remain engaged. 

More research is needed in the area of technology-assisted health lifestyle management, including qualitative research to more fully understand college students’ values, perceptions, needs, and preferences when it comes to the use of technologies. Finally, ongoing collaboration is needed between college administrators, student services staff, technology experts, and researchers to better understand what college students need to manage their health in quality ways, especially during the first years of adjustment to college life. Funders should be encouraged to continue supporting research in this area.

## Data Availability

The data collected during the two pilot studies are in the possession of the corresponding author.
